# Environments, processes, and outcomes - using the LEPO framework to examine medical student learning preferences with traditional and electronic resources

**DOI:** 10.1080/10872981.2021.1876316

**Published:** 2021-01-26

**Authors:** Kristin Wong, Vidhi Kapoor, Alan Tso, Mary OConnor, David Convissar, Neil Kothari, Christin Traba

**Affiliations:** a Departments of Medicine and Pediatrics, Rutgers New Jersey Medical School, Newark, NJ, USA; b Department of Medicine, University of Washington, Seattle, WA, USA; c Department of Anesthesiology, Harvard University, Cambridge, MA, USA; d Department of Medicine, Rutgers New Jersey Medical School, Newark, NJ, USA; e Department of Pediatrics, Rutgers New Jersey Medical School, Newark, NJ, USA

**Keywords:** Learning preferences, medical students, traditional resources, third-party resources, learning environments, learning processes, learning outcomes, medical school curricula

## Abstract

Changes in medical student learning preferences help drive innovation in teaching and require schools and commercial resources to quickly adapt. However, few studies have detailed the relationship of learner preferences to the environment and teaching modalities used in the pre-clerkship years, nor do they incorporate third-party resources. Our study attempts to analyze learner preferences by comparing the use of traditional and third-party resources. In 2017–18, a survey was distributed to medical students and residents at two accredited medical schools. Participants noted preferred styles of learning regarding lecture duration, timing, location, format, third-party resources, learner types and USMLE Step 1 scores. The ‘Learning Environment, Learning Processes, and Learning Outcomes’ (LEPO) framework [5] was used to examine learner preferences, with responses compared using the Mann-Whitney U and two proportion z-tests. A total of 329 respondents completed the survey: 62.7% medical students and 37.3% residents. The majority of participants identified their learning style by Kolb [6] as converging (33.0%) or accommodating (39.2%). Students preferred lectures 30–40 minutes long (43.3%), during morning hours (54.2%), in their own homes (52.0%), via online lectures with simultaneous drawings (56.0%), and classroom/podcast lectures with PowerPoint® presentations (54.3%). Overall, students rated third-party resource characteristics higher than traditional curricula, including effectiveness of teachers, length, quality, time of day, and venue (p < 0.001), but also preferred small group formats. Students reported animated videos (46.6%) and simultaneous drawings (46.5%) as the most effective means of retaining information. Understanding changing learner preferences is important in creating optimal curricula for today’s students. Using the LEPO framework, this study identifies critical preferences in successfully teaching medical students, inclusive of commercial and traditional resources. These results can also help guide changes in pedagogy necessary due to the more recent COVID-19 pandemic.

## Introduction

Delivery of medical school education has been a constantly adapting process over the years. Prior to the establishment of formalized medical education in the early 19^th^ century, the primary form of learning and evaluation for medical students was solely based on individual apprenticeship [1]. Since the establishment of formalized medical schools, medical education strives to improve the methods and manner in which information is presented to medical students. There are sweeping advances in technology that propel our medical educators to adapt to their current times and student preferences.

The prevailing practice of preclerkship medical education delivery within several medical schools is in the form of lectures with an increasing proportion of team-based learning activities and small group case-based discussions. There have been several studies examining the retention of information from lecture materials that show information may be better retained with the use of more visual aids [2] and shorter duration of lectures [3], however, they do not assess the widespread use of commercial resources as a supplement to medical student study habits.

The variety of resources available to students, both traditional and electronic, have given them an opportunity to explore different methods of teaching and learning that offer a variety of learning styles beyond the traditional classroom lectures. There have been some international studies comparing the use of commercial or third-party resources and the study preferences of medical students [4], however, no such studies have been conducted in the USA. Given the significant differences in the structure of medical education worldwide, it is difficult to apply such results to US medical schools.

It is also important to analyze these resources using an educational framework and understand their impact on medical education. The ‘Learning Environment, Learning Processes, and Learning Outcomes’ (LEPO) framework proposed by Philip et. al. [5] reframes learning as a process and teaching as an activity, noting that the majority of learning occurs outside of formal lectures. The three components of learning include: 1) the learning environment (environment facilitating learning), 2) learning processes (activities), and 3) learning outcomes (knowledge, behavior, skills, understanding). By using this framework, our study aims to review the traditional methods of medical school instruction versus the use of commercial or third-party resources within the context of medical student learning preferences, including the learning environment, learning processes/activities, and learning outcomes. With a more robust understanding of student preferences and their study habits immediately pre-pandemic, medical schools can create more effective delivery models for their curriculum and determine which post-pandemic changes should be considered temporizing versus permanent.

## Methods

During the 2017–2018 academic year, medical students and residents at two Liaison Committee on Medical Education(LCME)-accredited institutions received an anonymous, multi-question survey via Qualtrics, a secure online software. The establishment’s Institutional Review Board (IRB) approved the expedited study.

The survey was disseminated once via email with monthly reminders over 3 months (November to January) to all students and residents. In addition to emails, students and residents were reminded to consider participation via verbal announcements at student lectures and resident conferences. As an incentive for participation, four, internally funded, 25.00 USD gift cards were offered to participants through a raffle at the end of the study. Subsequent statistical analysis was performed via Excel®. Mann-Whitney U tests and two proportion z-tests were utilized in comparing groups.

The survey contained 28 questions assessing participant demographics, USA Medical Licensing Examination (USMLE) Step 1 score, and their study preferences. Participants were also asked to assess their medical school curricula and third-party resources regarding lecture length, location, presentation of material, timing of lectures or resources used, and quality of different characteristics of curricula. Finally, participants were asked about their overall medical educational experiences regarding hours spent on each mode of material/presentation and estimated retention of medical knowledge for each of these modalities.

Our study chose to use the LEPO framework in the analysis and discussion of our findings. This framework as proposed by Philip et. al. takes a broader view at the interactions between learning environments, processes, and outcomes and adopts many different aspects of frameworks developed by Biggs, Laurillard, Bain, Reeves and Reeves, and Goodyear [5]. Philip et. al. also uses this framework to analyze the potential of e-learning, which in the current pandemic requires further elaboration and integration in medical education. Using the learner as the centerpiece in this study, our results elucidate how learners prefer to ‘work within’ the learning environment, how they prefer to ‘engage in’ the learning process, and how they ‘demonstrate’ learning outcomes [5].

## Results

There were 358 respondents that initiated the survey and 329 respondents completed it. All respondents were active medical students or residents at two LCME-accredited institutions during the 2017–2018 academic year. Most of the respondents were aged 23–30 years (72.3%, n = 259) and were attending or had attended one of the institutions (59.5%, n = 213). The other institution, other US allopathic medical schools, osteopathic schools, and international medical schools were also represented in the data (13.1%, 9.8%, 4.7%, and 12% respectively). There were more medical student participants (62.7%, n = 224) than resident participants (37.3%, n = 133) in this study. (Table 1)

**Table 1.** Demographics

**Age (years)**	**n = 358**	**%**
22 or younger	29	8.1%
23–30	259	72.3%
31 or older	70	19.6%
**Graduate Level**	**n = 357**	**%**
UME (Medical Students)	224	62.7%
GME (Residents/Fellows)	133	37.3%
**Medical Schools**	**n = 355**	**%**
US Allopathic	295	83.1%
International	43	12.1%
US Osteopathic	17	4.8%
**Learner Types**	**n = 324**	**%**
Accommodatingᵃ	127	39.2%
Convergingᵇ	107	33.0%
Assimilatingᶜ	52	16.0%
Divergingᵈ	38	11.7%
**USMLE Step 1 Scores**	**n = 233**	**%**
Less than 190	5	2.1%
191–210	33	14.2%
211–230	64	27.5%
231–250	89	38.2%
Greater than 250	42	18.0%

ᵃActive Processing & Concrete PerceivingᵇActive Processing & Abstract PerceivingᶜReflective Processing & Abstract PerceivingᵈReflective Processing & Concrete Perceiving

Participants were asked to select how they processed and perceived information which corresponded to particular ‘learning styles.’ Of the 4 learning styles (Diverging, Assimilating, Converging, and Accommodating) defined by Kolb [6], most respondents were of the converging (33.0%, n = 107) or accommodating (39.2%, n = 127) types. Over one third of respondents reported a USMLE Step 1 score within a range of 231–250 (38.2%, n = 89). (Table 1) There was no significant difference in USMLE scores among the different learning styles. Subgroups were small and subsequently no significant differences in learning preferences by demographics, learning styles, or USMLE Step score were appreciated.

**Figure 1.**
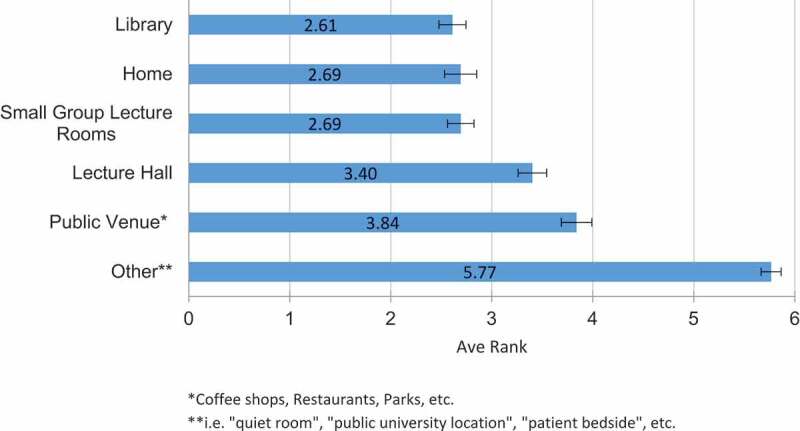
Preferred Study Locations by Average Rank

Participants also selected various factors in which they felt they ‘learned the best’ to assess their preferred study or learning condition – i.e. how they prefer to ‘work within’ the learning environment. Approximately half of participants reported that they ‘learned the best’ when lectures are 30–40 minutes long (43.3%, n = 155) and during morning hours between 8 AM and 12 noon (54.2%, n = 194). (Table 2) Of note, an error in the survey prevented students from selecting lecture lengths of 50–60 minutes long, however, there was still a statistically significant preference for lectures less than 40 minutes (74%, n = 265) than lectures greater than 40 minutes (26%, n = 93). Participants were also asked to rank in order of preference, 1 being the most preferred and 6 being the last, in which location do they study the best. Participants equally preferred studying in a library, small group lecture room, or at home (average rank 2.61, 2.69, 2.69, CI 0.134, 0.131, 0.159 respectively). (Figure 1)

**Table 2.** Comparison of Study Preferences with Medical Schools and Third-Party Resources

	**Student Preferences**		**Medical School Curricula Format**				**Third-Party Resources Format**			
**Lecture Length**	**choice count**	**% of respondents**	**choice count**	**% of respondents**	**z-score**	**p-value**	**choice count**	**% of respondents**	**z-score**	**p-value**
10–20 min	26	7.3%	1	0.3%	4.861	<.001	81	26.7%	−6.772	<.001
20–30 min	84	23.5%	3	0.9%	9.187	<.001	106	35.0%	−3.261	0.001
30–40 min	155	43.3%	0	0.0%	13.963	<.001	43	14.2%	8.140	<.001
40–50 min	87	24.3%	66	18.9%	1.799	0.072	18	5.9%	6.435	<.001
50–60 min*	–	–	247	70.6%	–	–	22	7.3%	–	–
1 hour or more	6	1.7%	33	9.4%	−4.502	<.001	33	10.9%	−5.010	<.001
**Time of Day**	**choice count**	**% of respondents**	**choice count**	**% of respondents**	**z-score**	**p-value**	**choice count**	**% of respondents**	**z-score**	**p-value**
Morning (8am-12pm)	194	54.2%	342	97.7%	−13.496	<.001	47	14.4%	11.444	<.001
Afternoon (12pm-5pm)	74	20.7%	6	1.7%	7.964	<.001	90	27.6%	−1.591	0.056
Evening (5pm-12am)	78	21.8%	2	0.6%	8.914	<.001	168	51.5%	−7.323	<.001
Other	12	3.4%	0	0.0%	3.455	0.001	21	6.4%	−1.671	0.475
**Study Location**	**1st or 2nd choice count**	**% of respondents**	**choice count**	**% of respondents**	**z-score**	**p-value**	**choice count**	**% of respondents**	**z-score**	**p-value**
Library	173	49.7%	138	39.4%	2.733	0.003	153	46.5%	0.835	0.203
Home	181	52.0%	201	57.4%	−1.438	0.075	263	79.9%	−7.645	<.001
Small Group Lecture Rooms	159	45.7%	98	28.0%	4.845	<.001	32	9.7%	10.392	<.001
Lecture Hall	94	27.0%	177	50.6%	−6.386	<.001	13	4.0%	8.221	<.001
Public Venue	76	21.8%	53	15.1%	2.279	0.011	96	29.2%	−2.193	0.014
Other	13	3.7%	5	1.4%	1.923	0.027	10	3.0%	0.500	0.309
**Presentation Styles**	**choice count**	**% of respondents**	**choice count**	**% of respondents**	**z-score**	**p-value**	**choice count**	**% of respondents**	**z-score**	**p-value**
Online lectures with simultaneous real time drawing/animations	200	56.0%	22	6.3%	14.226	<.001	203	62.3%	−1.658	0.097
Classroom or ‘podcasts’ of classroom lectures with PowerPoint®	194	54.3%	319	91.4%	−11.047	<.001	59	18.1%	9.797	<.001
Small Groups (12 or fewer students/group)	186	52.1%	167	47.9%	1.129	0.258	4	1.2%	14.820	<.001
Online Animated Videos	181	50.7%	8	2.3%	14.524	<.001	155	47.5%	0.824	0.412
Live interactive lectures with the use of chalkboard/whiteboards (i.e. chalk talks)	173	48.5%	74	21.2%	7.592	<.001	64	19.6%	7.905	<.001
Reading books or lecture notes	153	42.9%	74	21.2%	6.159	<.001	140	42.9%	−0.023	0.984
Large Group labs (i.e. physical exam session, etc.)	47	13.2%	143	41.0%	−8.330	<.001	3	0.9%	6.137	<.001
Lectures with no visuals (Audio/Speaking only)	15	4.2%	20	5.7%	−0.936	0.347	35	10.7%	−3.275	0.001

*Survey error – 50-60 min choice not available for student preferences. Overall, students preferred lectures <40 min (74%, n = 265), whereas 1.1% (n = 4, p < 0.001) of medical school lectures and 75.9% (n = 230, p = 0.575) of third-party lectures were <40 min.

When comparing student preferences to medical school and third-party formats, most medical school lectures were longer (>40 min, 98.9%, n = 346) while third-party lectures were shorter (<40 min, 75.9%, n = 230). Most medical school lectures were delivered in the morning, matching student preferences, but third-party resources were utilized in the evenings. Both medical school and third-party materials were mostly utilized at home (57.4% and 79.9% respectively). A two-proportion z-test was used to assess for significant differences in curricula compared with student preferences. Most medical school and third-party formats of curricula were statistically different from student preferences (p < 0.01). (Table 2)

**Figure 2.**
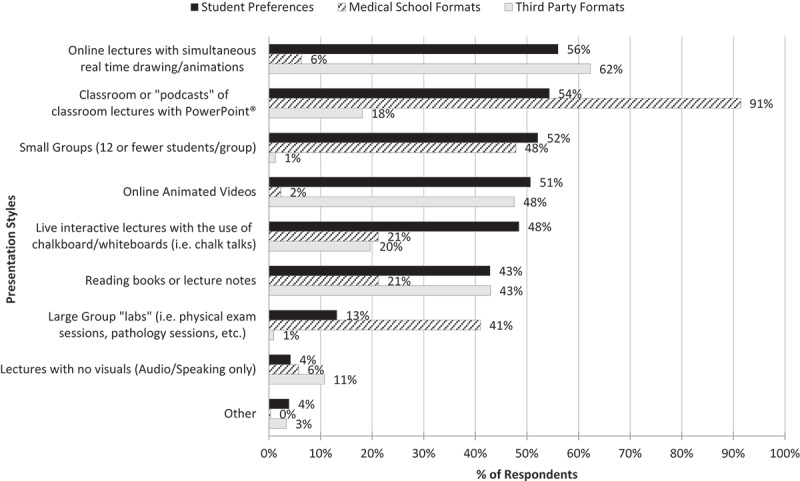
Comparison of Preferred Presentation Styles with Medical Schools and Third-Party Resources

Students were also asked about preferred elements in which they ‘engaged in’ the learning process. The least selected presentation styles when students were asked ‘how they learned the best’ were large groups and audio only type lectures (13.2%, n = 47 and 4.2%, n = 15 respectively), however, a large portion of students (41%, n = 143) had medical school curricula delivered via large groups. (Figure 2) More interactive presentation styles seemed to be preferred, which included classroom lectures with PowerPoint®, the most common style delivered by medical schools (91.4%, n = 319), and online lectures with animation, the most common style delivered by third-party resources (62.3%, n = 203). Students also preferred small group learning, which medical schools delivered but third-party resources did not, and online animated videos and review books which third-party resources delivered but medical schools did not. A two-proportion z-test again showed that there were statistically significant differences between student preferences and medical school or third-party provided styles for several types of presentations. (Table 2)

Characteristics of medical school and third-party resources were compared using a 5-point Likert scale from very poor (1 point) to very good (5 points). Students rated the third-party resources higher than the medical schools in terms of length of lecture (4.55 vs 3.27), effectiveness of teachers (4.75 vs 3.34), quality of lecture material (4.58 vs 3.31), time of day of lectures (4.5 vs 3.73), and venue of lectures (4.51 vs 3.85). Using the Mann-Whitney U test, there was a statistically significant difference (p < 0.001) in all characteristics (Table 3). Subgroup analyses by medical school and type also showed statistically lower ratings (p < 0.01) between medical school curricula and third-party resources in all characteristics and for all schools.

**Table 3.** Ratings (5-point Likert) of Curriculum Characteristics & Estimated Use & Efficiency of Curricular Material

**Characteristics of Curriculum**	**Avg Rating of Medical Schools**	**Avg Rating of Third-Party**	**z-score**	**p-value**
Length of Lecture	3.27	4.55	−15.692	<.001
Effectiveness of Teachers	3.34	4.75	−16.556	<.001
Quality of Lecture Material (Handouts, PowerPoints, videos, notes, etc.)	3.30	4.58	−15.248	<.001
Time of Day of Lectures	3.73	4.51	−9.429	<.001
Venue of Lectures (i.e. classroom, lecture hall, at home, etc.)	3.85	4.51	−8.837	<.001
**Estimated Use & Efficiency of Curricular Material**	**Avg Hr/Day**	**Avg % Retained**	**% Retained/Hr**	**Source of Med Knowledge %**
Classroom Lectures/Podcasts	2.68	26.0	9.7	24.0
Live interactive lectures with use of chalkboard/whiteboard (i.e. ‘chalk talks’)	0.86	34.8	40.7	5.8
Online lectures with simultaneous real time drawing/animations	1.02	46.5	45.6	11.8
Online animated videos	0.93	46.6	50.1	7.8
Lectures with no visuals (Audio/Speaking only)	0.45	15.7	34.6	1.4
Small Groups (12 or fewer students/group)	1.31	33.3	25.3	6.1
Large Groups or ‘labs’ (i.e. physical exam sessions, pathology sessions, etc.)	1.25	23.7	19.0	3.6
Reading school notes or textbooks	2.08	37.0	17.8	13.5
Reading review books (i.e. First Aid, Case Files, etc.)	1.82	44.2	24.3	17.5

Lastly, our study tried to capture how learners demonstrate learning outcomes. Respondents were asked to estimate the number of hours spent per day with various types of curricular modalities and how much of the material they believe they retained from each. By correlating the average time spent with the average percent retained, we estimated each modality’s efficiency for learning. This was then compared with the breakdown of where respondents received their medical knowledge overall. Animated videos and online lectures with live animation were calculated as the most efficient forms of material for learning (50.1% retained/hour and 45.6% retained/hour respectively). However, most respondents received their medical knowledge through classroom lectures (24%), which seemed to be the least efficient means of learning (9.7% retained/hour). (Table 3)

## Discussion

The COVID-19 pandemic has forced schools to create innovative ways to provide distance learning to all students in the name of social distancing and safety. Some question whether these changes should be permanent or just temporary, leading to the importance of a robust understanding of student preferences prior to making permanent changes. Philip et. al.’s LEPO framework provides a broad context in which to review and discuss the results of this survey.

### Learning environment

With an acknowledgement of the ‘learning environment,’ traditional and electronic, the LEPO framework begs for informal and detailed reviews of learner characteristics which influences learning processes and is determined by learning outcomes [5]. Below is a detailed review of how learners work within the learning environment as revealed by our study.

#### Length of lecture time

In our results, there was a split between the preferred length of lectures and the length of lectures offered. While 43.3% of our respondents reported that they preferred lectures that are 30–40 minutes long, most lectures are currently 50–60 minutes in length; 26% preferred lecture lengths longer than 40 minutes, out of which only 1.7% of the participants preferred lectures that were 1-hour or longer. This was further reflected in the quality assessment of curricular aspects where only 45% of students found the lecture lengths to be ‘acceptable.’ In the larger scheme, these results are consistent with prior studies demonstrating that the preferred length of lecture time is 30 minutes with the highest information recall being between the fifteenth and thirtieth interval [2]. Even more, we know that many of the popular, commercially available resources utilized by medical students offer 20 to 30-minute lectures which further verifies that the current medical learners’ preference is for shorter lectures.

#### Time of day for learning

Students stated that morning time is the most effective for learning via lectures. We did not specifically investigate medical students’ ideal lecture start time. However, in 2014, the American Academy of Pediatrics issued a policy statement recommending school start times after 8:30am [7]. This was based on studies, which showed that delaying school start times is an effective countermeasure to chronic sleep loss and has a wide range of potential benefits regarding their physical and mental health, safety, and academic achievement. The American College Health Association National College Health Assessment II executive summary found that 24.8% of students reported that sleepiness during daytime activities is more than a little problem and 12% reported that it was a big problem [8]. Other countries have also found an association of daytime sleepiness and academic performance in medical students [9,10].

Despite these findings, there are limited studies on sleep disorders/habits in US medical students. Ayala et al found that sleep qualities were poor among first and third-year medical students as well as minorities [11]. It is likely that medical students’ study and sleep habits do not change dramatically from their college experience though their study load may dramatically increase, leading to detriments in their learning. Although learners preferred their lectures to be in the mornings, our study showed that they also participated in evening self-study. We did not investigate specific times such as intervals/length of evening self-study and actual bedtimes, but further investigation on the relationship between medical learners’ sleep and study habits would aid medical schools to strategize their lecture start times to enhance learning during the preclerkship years of medical school.

#### Preferred locations

While there have been a few studies examining optimal learning spaces for students such as active learning classrooms and the layout of library spaces, there is a dearth of literature examining the study spaces preferred by US medical students [12,13]. Based on our results, the most popular study spaces preferred and utilized by medical students were the library, home, and small group lecture rooms with 52% of respondents ranking their home as their first or second choice for learning. (Table 2) Given the COVID-19 pandemic and the necessity for social distancing, medical schools are expecting curricula to be used more in private spaces, possibly aiding in student study habits. This dramatic change in environment by which students access preclerkship education, should be strongly considered when determining the format and structure of curricula, accounting for the percentage of traditional large lecture hall didactics needed after the pandemic.

The least preferred learning spaces were lecture halls and public venues. Prior to the pandemic, medical schools frequently delivered curricula via large lecture halls, demonstrating a divide between medical student preferences and the study spaces they spend a majority of their time in. There was also a difference in 45.7% of respondents preferring to study in small group lecture rooms, but only 28% of respondents use these rooms in the medical schools. Some studies have examined the role and attributes of spaces best suited for a productive learning environment; a merger of learning theory with the physical aspects of the architecture are thought to produce the most efficient learning spaces for students [14]. A study conducted at Nanyang Technological University in Singapore examined the utilization of informal learning spaces by undergraduate students [15]. They concluded that student preferences for learning spaces were based on three main characteristics or ‘3 C’s’: 1) comfort of the learning environment (furniture, temperature, cleanliness, internet connections etc.), 2) the convenience of the space (accessible locations closer to classes or any restrictions on consumption of foods and drinks, etc.), and 3) a sense of community that balances private space as well as spaces enabling group collaboration [15]. A similar approach can be taken for designing learning spaces for students and residents in the medical community to ensure availability of preferred spaces amid time constraints and heavy study loads. Now with medical schools increasingly adopting distance learning methods, students may benefit from being able to access curricula from home where students can adapt all ‘3 C’s’ to their own preferences.

### Learning processes

The LEPO framework also calls for the analysis of how learner preferences or characteristics play a role in the learning process or activities. These activities subsequently lead to learning outcomes and serve as examples in how learners engage with a curriculum [5]. As our study focuses on how the learner interacts within the LEPO framework, below we detail aspects of how learners prefer to engage in the learning process.

#### Preferred modality

Traditionally, the primary method of delivering medical school curricula has been through classroom or podcast lectures with PowerPoint® (91.4%), however, in this study, we found preferences for other formats. (Figure 2) Other preferred formats seemed to have more visually stimulating aspects like in the form of animated videos or real time drawings or ‘chalk talks.’ In our study, most of the participants’ preferred learning style was to process information actively vs reflectively (73.1%, n = 256 vs 26.9%, n = 94). There seemed to be an even distribution of how participants preferred to perceive information, either abstractly or concretely (49.7%, n = 165 vs 50.3%, n = 167 respectively). While active learning styles do not necessarily correspond to visual learning, it does seem that visually stimulating material is more interactive and therefore a preferred method for active learner types. More visually stimulating material also seemed to be the most efficient means of studying as noted in Table 3. These observations seem to be consistent with studies comparing retention of information presented visually versus verbally, noting that visual information was far superior at short-term and long-term recall2. Although PowerPoint® lectures have been successfully utilized in many different educational curricula, given the amount and depth of information medical students are taught, medical school curricula may be more effectively delivered by incorporating more animation in the lectures.

#### Size of learner groups

Decades of research in learning have demonstrated the positive outcomes with collaborative learning [16]. Our cohort was familiar and had success with these learning methods while in college. This may explain their preference to small group learning versus lecture-based learning. Another learning modality that has gained popularity in undergraduate learning is the flipped classroom model. In this model, students are provided online learning materials before the small group discussion. The idea is to promote active learning and collaborative learning. In 2017, Ramnanan demonstrated that undergraduate medical students had a strong preference for flipped classroom models over the traditional lectures [17]. Our study seemed to support these preferences, with 52.1% of respondents preferring small group didactics. Only 13.2% preferred didactics via large groups or ‘labs.’ Respondents also seemed to find small group learning as efficient as reading review books. (Table 3) As distance learning becomes more prevalent, medical schools may want to reincorporate ‘small group’ didactics after social distancing requirements are lifted, especially given student preferences and effectiveness of learning.

#### Third-party resources

In general, most medical students and residents use third-party resources. Based on survey results, third-party resources tend to provide more animated or visually stimulating material in short bursts of time. These formats did seem to result in more ‘learning efficiency.’ However, third-party resources fell short in providing other interactive styles like small group didactics. While the quality assessment of the curricular aspects clearly favored third-party resources, it should be noted that students likely purchase additional resources based on their preconceived preferences and quality of material, therefore biasing any direct comparisons. Still, while medical schools evaluate their curricular modalities, being sure to reinvest in teachers of high quality, providing more visually stimulating material, and providing flexible means of access by time and location will likely significantly impact a student’s impression on the quality of their education.

### Learning outcomes

It is interesting to note that while students seem to have more learning efficiency with online animated videos, other electronically available means of learning also showed significant learning efficiencies over more traditional means of learning like textbooks and large group labs. In discussing the use of the LEPO framework, Philip et. al. pays tribute to the complexity of e-learning and evaluating its effectiveness. Like traditional means of learning, electronic means carry complex relationships with learners, teachers, environments, processes, and outcomes [5]. However, based on our study results, electronic means of learning may not be as risky to the quality of education as some may be wary of and perhaps provides some encouragement to the forced curricular changes currently happening at medical schools because of the pandemic.

In addition, while classroom lectures/podcasts appear to be the least efficient means of learning, the magnitude and frequency of its use carries a significant impact on learners as a whole. On the surface, this appears to be a monstrous dilemma in providing traditional medical school curricula, however, simply adding animation or chalkboard/whiteboard-based visual cues within the lectures can suddenly turn podcasts into the most preferred and efficient means of learning. In this regard, medical schools can take solace in targeting such low hanging fruit in the design of their curricula.

#### Limitations

While the survey included two, independent, LCME-accredited institutions and captured a diverse cadre of residents from various medical schools, the data largely represented student preferences at one institution. There were some limitations in the available answer choices, as noted in the lecture length choices, and the ability to make comparisons between survey responses due to the use of categorical data versus continuous data. Estimating the retention and sources of medical knowledge was also limited as they were self-reported numbers – objectively testing retention after using various presentation styles would be more accurate, but observation-based studies would need to account for any Hawthorne effects. Further, given the varying education level of respondents, students and residents were rating aspects of their medical school’s curriculum as well as third-party resources at different intervals of time from when they each experienced the preclerkship curriculum.

It is also important to recognize that this study keeps the learner at its core and should be acknowledged that further studies need to focus on the teacher as the other critical component in education as posited by Philip et. al [5]. The interrelatedness of teachers within the learning environment, learning processes, learning outcomes and to learners directly, cannot be understated, but would require further study of their role as it is affected by the current pandemic.

## Conclusion

Over the past years, the medical community has remained open to trialing new methods of implementing medical school curricula. As medical schools around the country are forced to adopt virtual methods of teaching due to the COVID-19 pandemic, our study identifies important learning preferences of students and highlights aspects of the medical education experience from the learners’ perspectives pre-pandemic. This understanding will help institutions decide which changes in teaching and learning environments should be considered temporary due to social distancing requirements or be made more permanent. Using the LEPO framework and detailing the interrelatedness of our learners with the environment, processes, and outcomes, we hope that the results of this study provide the medical community with data toward reforming certain aspects of medical education to enhance learner experiences and academic achievements. Making small adjustments to medical school curricula, such as the length of each lecture, can make a large impact on a student’s perception and integration of the material. Schools should consider adapting to learner preferences and more efficient means of learning by shortening their lecture times and providing more visually stimulating or animated material. Certain aspects of medical education seemed to be on target with student preferences like holding lectures in the morning and providing small group didactics, however, there needs to be further studies to assess for retention of medical knowledge and review the impact of these changes to medical school curricula.

## References

[1] Kendall PL, Reader G. Innovations in Medical Education of the 1950’s Contrasted with Those of the 1970’s and 1980’s. *J* *Health Soc Behav*. 1988;29(4):279. 3075629

[2] Giles R, Johnson MR, Knight KE, et al. Recall of lecture information: a question of what, when and where. *Med Educ*. 1982;16(5):264–9. 7132803 10.1111/j.1365-2923.1982.tb01262.x

[3] Stuart J, Rutherford R. Medical student concentration during lectures. *Lancet*. 1978;312(8088):514–516. 10.1016/s0140-6736(78)92233-x 79879

[4] Holambe V, Thakur NA, Giri PA. Student’s preferences for learning in medical education. *Int* *J* *Community Med Public Health*. 2015;2:328–330.

[5] Phillips R, McNaught C, Kennedy G. (2011). The Learning Environment, Learning Processes and Learning Outcomes (LEPO) Framework. In: *Evaluating e-Learning* (pp. 23-42). New York, NY: Routledge.

[6] Kolb AY, Kolb D. (2005) The Kolb Learning Style Inventory - version 3.1. In: *2005* *Technical specifications (pp. 1-71)*. Boston, MA: Hay Resources Direct.

[7] Au R,Carskadon M, Millman R, et al. School Start Times for Adolescents. *Pediatrics*. 2014;134(3):642–649. 25156998 10.1542/peds.2014-1697 PMC8194457

[8] Association ACH. National college health assessment II: reference group executive summary spring 2019. In: *American College Health Association*. Silver Spring, MD; 2019.

[9] Abdulghani HM, Alrowais NA, Bin-Saad NS, et al., Sleep disorder among medical students: relationship to their academic performance. *Med Teach*. 2012;**34**(s1(sup1):S37–S41. 22409189 10.3109/0142159X.2012.656749

[10] Rodrigues RND, Viegas, CA, Silva AAA, et al. Daytime sleepiness academic performance in medical students. *Avg Neuropsiquiatr*. 2002;60(1):6–11. 10.1590/s0004-282x2002000100002 11965401

[11] Ayala EE, Berry, R., Winseman JS, et al. A Cross-sectional Snapshot of Sleep Quality and Quantity among US Medical Students. *Acad Psychiatry*. 2017;41(5):664–668. 28091977 10.1007/s40596-016-0653-5

[12] Cunningham HV, Tabur S. Learning space attributes: reflections on academic library design and its use. *Journal of Learning Spaces*. 2012;1(2):1-6.

[13] Talbert R, Mor-Avi A. A space for learning: an analysis of research on active learning spaces. *Heliyon*. 2019;5(12):e02967. 32368631 10.1016/j.heliyon.2019.e02967 PMC7190693

[14] Painter S, Fornier J, Grape C, et al. (2013). *Research on learning space design: present state, future directions*. e-book:Society for College and University Planning. www.scup.org/perrychapman.

[15] Lee JWY. (2017). Learning spaces around the university: factors that affect the preference for a space. In: *3rd International Conference on Higher Education Advances (pp. 382-390)*. Val`encia: Universitat Politecnica de Val`encia.

[16] Scager K, Boonstra J, Peeters T, et al. Collaborative learning in higher education: evoking positive Interdependence. *CBE Life Sci Educ*. 2016;15(4):ar69. 27909019 10.1187/cbe.16-07-0219 PMC5132366

[17] Ramnanan CJ,PL. Advances in medical education and practice: student perceptions of the flipped classroom. *Adv Med Educ Pract*. 2017;8:63‐73. 10.2147/AMEP.S109037 PMC5245805 28144171

